# Cost-benefit analysis of on-farm grain storage hermetic bags among small-scale maize growers in northwestern Ethiopia

**DOI:** 10.1016/j.cropro.2020.105478

**Published:** 2021-05

**Authors:** Gashaw Tenna Alemu, Zerihun Nigussie, Nigussie Haregeweyn, Zewdu Berhanie, Beneberu Assefa Wondimagegnehu, Zemen Ayalew, Dessalegn Molla, Eric Ndemo Okoyo, Dieudonne Baributsa

**Affiliations:** a The United Graduate School of Agricultural Sciences, Tottori University, 4-101 Koyama-Minami, Tottori, 680-8553, Japan; b Arid Land Research Center, Tottori University, 1390 Hamasaka, Tottori, 680-0001, Japan; c College of Agriculture and Environmental Sciences, Bahir Dar University, P.O. Box 5501, Bahir Dar, Ethiopia; d International Platform for Dryland Research and Education, Tottori University, 1390 Hamasaka, Tottori, 680-0001, Japan; e GIZ (Deutsche Gesellschaft für Internationale Zusammenarbeit) Office, P.O. Box 100009, Addis Ababa, Ethiopia; f College of Agriculture and Environmental Sciences, Haramaya University, P.O. Box 138, Dire Dawa, Ethiopia; g Department of Entomology, Purdue University, West Lafayette, IN, 47907, USA

**Keywords:** Drought, PICS, Postharvest management, Profitability, Gross margin

## Abstract

Farmers continue losing substantial quantities of grain during storage due to damages from pests including insects. Hermetic bags, being promoted in Ethiopia, could be viable alternatives to traditional methods and insecticides that are commonly used by farmers to store grain. However, the economics and determinants behind farmers' decisions to use different storage methods are poorly understood. This study sought to ascertain the economics of hermetic grain storage technology among 450 representative small-scale maize farmers in northwestern Ethiopia. Gross margin (GM), and the marginal rate of return (MRR) were employed to estimate the economic costs and benefits of storage methods, while a multivariate probit regression model was employed to analyze the determinants of farmers' decision to store maize with a given storage method. The results show that farmers used a combination of different storage techniques: 19.6% did not store grain, 87.8% used traditional methods with pesticide, and 66.7% used Purdue Improved Crop Storage (PICS) hermetic bags. Farmers who used hermetic bags also used other mentioned storage techniques. PICS had the highest GM (US$21.77 100 kg^−1^) and MRR (3.196), indicating that they were the most profitable. Moreover, a household could obtain an additional net cash flow of US$5.02 100 kg^−1^ PICS bag per season after 9.6 months of storage. Farmers’ decisions to use PICS bags were influenced by several factors including access to information, the initial cost, and storage capacity of the technology. Thus, increasing awareness and improving supply chain efficiency to reduce the cost of the PICS bags would improve adoption rates.

## Highlights

• The PICS bag has been widely promoted to reduce storage loss in Ethiopia, but its economic viability are poorly studied.
• The cost and benefit of using PICS bags for maize storage in northwestern Ethiopia were analyzed in this study.
• PICS bags had the highest gross margin (US$21.77 100 kg^−1^) among storage options.
• Households' decision to adopt PICS bags were influenced by the initial cost of the technology, storage capacity, and access to information.
• Increasing awareness and improving the supply chain efficiency to reduce cost would improve adoption rates.

## 1 Introduction

Sub-Saharan Africa (SSA) produces more than 112-billion-kg year^−1^ of grains for home consumption and as a source of income for millions of households (World Bank, 2011). Maize (*Zea mays* L.) is an important cereal crop in Ethiopia grown by over 11.5 million smallholder farmers with an average farm size of 2.27 ha (FAO, 2018). The average maize yield in Ethiopia are estimated at 3292 kg ha^−1^ (CSA, 2020), which is about half of the world's average yield (5924 kg ha^−1^) (World Bank, 2011). Lower yields combined with significant losses in the early stages of postharvest storage have made agricultural per capita returns low in SSA including Ethiopia (FARA, 2008; World Bank, 2011). Postharvest loss for maize in SSA due to storage insect pests is estimated to range between 10% and 20% (World Bank, 2011), while in Ethiopia, it is estimated between 5 and 26% (Befikadu, 2014; Hengsdijk and De Boer, 2017).

The storage of grains is critical for food supply and seed availability for small-scale farmers (Adetunji, 2009a). Lower quality grain due to insect infestation and high moisture content can significantly affect household food security, farmer income, and market price fluctuations. Improved storage technology could reduce postharvest loss for most cereal grains that are seasonally cultivated, and are subject to crop failure due to drought and other natural calamities (FAO, 1994). In SSA, popular storage types include traditional methods (e.g., granaries, underground storage, and clay pots), semi-modern methods (e.g., improved cribs), and modern methods (e.g., hermetic technologies, silos, insecticides, etc.) of which the first and the second are the most common (Udoh et al., 2000).

The major drawback of typical traditional storage methods is that the stored product is prone to attack by pests due to environmental conditions that favor insect development (Murdock et al., 2003). Traditional storage methods provide optimal conditions for pest infestation of stored grains (Ngamo, 2000; Adejumo and Raji, 2007). Smallholder farmers usually rely on chemicals to protect grains from storage pests (Baributsa et al., 2010). However, applications of these chemicals by farmers, affected by availability and access, vary from country to country within SSA. For instance, case studies found that, chemical use in maize storage by smallholder farmers accounted for 70% in Ethiopia (Tadesse and Basedow, 2004), 50% in Benin (Hell et al., 2000), 23% in Cameroon (Nukenine et al., 2002) and 12% in Eritrea (Haile, 2006).

Short-term and inter-seasonal market price fluctuations are common in SSA and affect both farmers and consumers. Most storage structures used in SSA have low gross margins (GMs) (i.e., low storage net value) because of the high total variable cost (TVC) incurred during the storage period. This low GM means that farmers in SSA lose a significant amount of the monetary value of their products by using traditional rather than improved storage techniques. As an example, maize that sells for US$0.084 kg^−1^ after harvest in February to March could realize US$0.158 kg^−1^ if stored until August to October in SSA (Grace et al., 2015). In Nigeria, farmers who used an improved maize storage technique (e.g., metal silos) had a GM of US$0.51 kg^−1^, whereas those who used a traditional storage method earned only US$0.23 kg^−1^ (Oladejo, 2016). Moreover, a cost-return analysis showed that using metal silos enabled farmers in Nigeria to achieve a gain in GM of US$0.06 kg^−1^ to US$0.09 kg^−1^ and a marginal rate of return (MRR) of up to 1.10 in comparison to other storage methods (Adetunji, 2009b). Additionally, the GMs for maize growers using various storage technologies were US$0.03 kg^−1^ (no storage), US$0.06 kg^−1^ (local), and US$0.09 kg^−1^ (modern) (Sekumade and Oluwatayo, 2009). In general, gross income losses occur when farmers have limited access to effective and safe storage technologies, such as airtight storage bags or metal silos (Jones et al., 2011; Gitonga et al., 2015).

Though these storage techniques have tremendous potential to improve household income and food security while reducing poverty (Tesfaye and Tirivayi, 2018; Brander et al., 2020; Chuma et al., 2020), their economic viability in rural settings of Ethiopia is not well investigated. Therefore, the objectives of this study were to (a) estimate the economic costs and benefits of the existing maize storage techniques and (b) analyze factors influencing maize growing farmers' decisions to use them in northwestern Ethiopia. The results of this study would provide quantifiable evidence on economic benefits, if any, that could be derived from the use of alternative storage technologies. Moreover, it will help to determine factors that would motivate the wider adoption of PICS.

## 2 Theoretical framework

In the study of economics of new agricultural innovations, farmers are expected to use the available innovation which has the highest benefits and lowest cost implications (Nigussie et al., 2020). For this study, farmers are considered as a consumer of the storage techniques, and there is a need to explain their preferences as a consumer. To explain consumer preferences on using maize storage techniques, it is logical to refer to a random utility framework (Lancaster, 1966). Based on this, when rational households faced with a choice among a set of feasible alternatives, such as choice of storage techniques, they are expected to choose the alternative which has the highest benefit. Hence, a household *n* would select storage technique *i* from a set of maize storage alternatives only if this alternative has the highest net expected benefit or utility compared to others, *j,* i.e. $E\left(U_{ni}\right)>E\left(U_{nj}\right)$.

Random utility models presume that the utility $U_{ni}$ derived by households from using the improved maize storage technology is composed of a deterministic component $V_{ni}$, and a stochastic error component $\varepsilon_{ni}$, which is the unobservable components influencing the choice such that:

$$U_{ni}=V_{ni}+\varepsilon_{ni}$$

The conditional probability P of using a given storage technology by a household based on the observable characteristics can then be estimated using the following:

$$P_{ni}=P\left(U_{ni}\right)>P\left(U_{nj}\right)\;for\;all\;j\neq i$$

## 3 Material and methods

### 3.1 Description of the study area

**Fig. 1:**
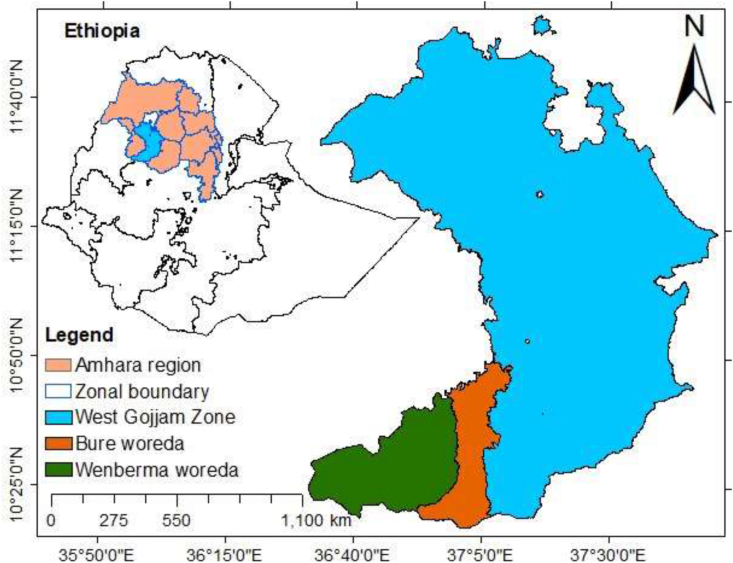
Map showing Ethiopia (left) and the study area (right).

West Gojjam Zone is located in the Amhara National Regional State (ANRS) of northwestern Ethiopia (Fig. 1). The population of West Gojjam is over 2.7 million with the total area of 13 312 km^2^. There are about 500 000 households, with an average of about 5.2 persons per household (ANRSPC, 2020). The main economic activity is agriculture, primarily crops (cereals, spices, pulses, vegetables, and oil) and livestock production. The area has been known for its high potential to supply maize, wheat, teff, and pepper (WGZDA, 2016). Womberma and Bure (study areas) are the leading maize-producing woredas (district) in ANRS (CSA, 2020). Farmers in the study area store maize using traditional techniques, pesticides, and improved techniques such as PICS bags (ANRSBoA, 2016). PICS bags are simple, chemical free, and triple-layer airtight that are used to store different types of grains (e.g., maize, cowpea, common beans, sorghum, rice, groundnut, and wheat) (Baoua et al., 2014; Baributsa et al., 2014; Baribusta et al., 2019).

### 3.2 Sampling design

PICS bags have been promoted and commercialized in the ANRS over the past four years due to the high maize production and potential interest in the technology by smallholder farmers. Sample respondents were selected based on a two-stage random sampling technique. First, three Peasant Kebele (ward) Administrations (PKAs) from each woreda were purposely selected: Zalma, Gulum Denjin, and Fetam Sentom in Bure woreda and Wogedad, Marquma, and Yergin in Womberma woreda. The selected PKAs have relatively higher maize production potential and number of PICS users than other adjacent PKAs. Second, simple random sampling was used to select 450 households from the selected PKAs. The list of households was obtained from local agriculture offices in each woreda. The fieldwork was undertaken between April and June 2017.

### 3.3 Data collection

Primary data were directly collected from respondents through informal and formal surveys. The preliminary informal survey was implemented in two phases. In Phase I, semi-structured interviews and key informant (KI) interviews were conducted to collect background data about maize storage techniques in the study area. The semi-structured interviews were conducted with 21 farmers following a checklist in a guided discussion. The KI interviewees included PICS suppliers, local dealers, extension agents, and other experts. In Phase II, Focus Group Discussions (FGDs) were held to gain preliminary information about the study area and a better understanding of local maize production, utilization, marketing, post-harvest management and extension information sources used by smallholder farmers. Insights gained from those informal interviews and discussion, coupled with our review of previous studies, enabled us to develop the interview questions to be used in the formal interviews. Following this step, a draft survey questionnaire was designed. Total of ten FGDs were conducted in the two woredas and 30 KIs were interviewed. Finally, the formal interviews of 450 respondents were conducted using a series of open- and closed-ended questions on the costs and economic benefits of the different storage methods used, and why they were being used. The specific types of data collected were: (a) socioeconomic characteristics of respondents, (b) types of maize storage techniques, (c) costs and economic benefits of maize storage techniques, and (d) farmers perceptions and reasons of using maize storage techniques. Relevant secondary data about market prices, production volume, costs and economic benefits were also collected from woreda and regional agricultural office reports, manuals, published books and research articles.

### 3.4 Data analysis

#### 3.4.1 Overview

Descriptive statistics were used to examine socioeconomic characteristics, existing maize storage techniques, average storage duration, and households reporting postharvest losses. The existing maize storage options in the study area were no storage or immediate sale after harvest (NS), traditional storage with pesticide (TSP), and PICS. Qualitative data from FGDs and KI interviews were also interpreted and described narratively to supplement the findings of the quantitative data analysis. Quantitative data management and analyses were performed using Stata ver. 15.1 (Stata Corp LP, College Station, TX, USA).

#### 3.4.2 Profitability analysis

For this analysis, households' self-reported economic costs and benefits associated with maize storage techniques were evaluated. In general, the decision to store is advisable when post storage benefit surpasses gross expenditures required for storage (Tierney et al., 1999). To determine the net value of grains stored using different methods, households’ self-reported primary data about the variable costs of storage were obtained. The difference of market value (price) of maize grain at several selling times was considered as an opportunity cost of each storage method and incorporated into the selling prices in the GM calculation. The costs that vary for each storage technique were included in the estimation of the net benefits of the storage method (Shepherd, 1999). Here, it must be clear that we included the variable cost items that would have differences related to each storage technique. Moreover, since all groups were maize producers and used local markets, there were no differences expected on the GM among storage techniques due to location and marketing expertise skills among the surveyed respondents. Storage material cost refers to a cost incurred to buy and/or construct a storage facility either from the market or from locally available materials. Usually, bags called *madaberia*,^1^ *akumada*,^2^ or *kiesha*^3^ in the local language are available in the local market for NS and TSP, whereas PICS bags are currently manufactured in the capital city Addis Ababa (the capital city of Ethiopia) and distributed across the country through a network of vendors. Although PICS has different packaging sizes (e.g., 25 kg and 50 kg) (Baribusta et al., 2019), the 100 kg bag was the only marketed and commonly used by farmers during the time of this study. Additionally, the study used average material cost because there is annual variation in the price of PICS bags. The chemical cost is the average cost incurred to buy and use pesticides when farmers store maize; this cost only applies to TSP because the other storage techniques do not require pesticides. The transportation cost is the average cost incurred for transporting maize from the home to the market place, and mule and/or donkey-driven carts were used for this purpose. The cost of time wastage is the economic cost incurred by the household while selling the product. It was calculated by the number of working hours in a day that a household lost for selling grain in the market multiplied by the average wage rate per hour in the locality. The cost of dry weight loss is the average economic cost as a result of the loss of moisture content during storage, and dry weight loss increases due to insect pests with longer storage durations under normal conditions. The cost of storage pest damage is the difference between values of insect damaged grain and undamaged grain. It was estimated from the responses of FGDs and KI participants, and it only applies to TSP.

The main output of this study was to compare the net value gained from each maize storage investment at the household level. One of the tools to compare economic benefits of technologies is partial budgeting (Lessley et al., 1991). Main et al. (2005) used partial budget analysis by using MRR to identify effective maize weed management options.

Partial budgeting compares the cost and benefits of different choices by estimating the difference in gains or costs expected from them (Eckersley, 2004). Lessley et al. (1991) pointed out the four major components of partial budget: added income (AI), decreased cost (DC), decreased income (DI), and added cost (AC). Accordingly, partial budgeting could be specified as:

(1) $$\mathrm{Positive}\;\mathrm{Effect}:\;\text{AI}+\text{DC}=P_{2j}Q_{2j}+r_{1j}X_{1j}$$

(2) $$\mathrm{Negative}\;\mathrm{Effect}:\;\text{DI}+\text{AC}=P_{1j}Q_{1j}+r_{2j}X_{2j}$$

(3) $$\mathrm{Difference}:\;\left(P_{2j}Q_{2j}+r_{1j}X_{1j}\right)-\left(P_{1j}Q_{1j}+r_{2j}X_{2j}\right)$$

where: AI is the added returns of the new technique, DC is decreased cost of the existing technique, DI is decreased returns of the existing technique, AC is the added cost of the new technique, $P_{2j}$ is price (US$) of a maize grain in the new storage technique for respondent *j*, $P_{1j}$ is price (US$) of a maize grain in the existing storage technique for respondent *j,* $Q_{2j}$ is quantity (kg) of maize grain in the new storage technique for respondent *j*, $Q_{1j}$ is quantity (kg) of maize grain in the existing storage technique for respondent *j*, $r_{1j}$ is price (US$) of variable input in the existing storage technique for respondent *j*, $r_{2j}$ is price (US$) of variable input in the new storage technique for respondent *j*, $X_{1j}$ is quantity (kg) of variable input in the existing storage technique for the respondent *j*, and $X_{2j}$ is quantity (kg) of variable input in the new storage technique for the respondent *j*.

The marginal analysis helps to sortout the potential returns of a change in using different maize storage techniques. It entails the derivation of MRR between techniques, where MRR is the proportion of GM deviations to the TVC deviations among different methods (Eckersley, 2004). Thus, it could be specified as:

(4) $$MRR=\frac{GM_{2}-GM_{1}}{TVC_{2}-TVC_{1}}$$

(5) $$MRR=\frac{\left[\sum_{i=2}\left(P_{2j}Q_{2j}-r_{2j}X_{2j}\right)\right]-\left[\sum_{i=1}\left(P_{1j}Q_{1j}-r_{1j}X_{1j}\right)\right]}{\sum_{i=2}r_{2j}X_{2j}-\sum_{i=1}r_{1j}X_{1j}}$$

The GM of a storage technique refers to the (stored) maize grain sales revenue a household could gain after incurring the variable costs associated with storing and selling to the market. Thus, it would then have specified as:

(6) $$GM=\overset{n}{\underset{ij}{\sum }}\left(P_{ij}Q_{ij}-r_{ij}X_{ij}\right)\text{,}$$

where: GM is gross margin (US$ 100 kg^−1^), $P_{ij}$ is price (US$) of a maize grain in storage technique *i* for respondent *j*, $Q_{ij}$ is quantity (kg) of maize grain in storage technique *i* for respondent *j*, $r_{ij}$ is price (US$) of variable input in storage technique *i* for respondent *j*, $X_{ij}$ is quantity (kg) of variable input in storage technique *i* for the respondent *j*, I = 1 … *p*, and *j* = 1 … *n*.

After deriving the GM, we used a compound interest rate to calculate the opportunity cost of capital (OCC) to normalize the differences of the lengths of storage duration among the storage techniques, i. e, 5.4 and 9.6 months of storage duration for TSP and PICS, respectively

(7) $$GM_{y}=GM_{x}{\left(1+\frac{r}{c}\right)}^{ct}$$

where: $GM_{y}$ is the final GM, $GM_{x}$ is the initial GM, r is the annual interest rate (i.e. 13.5% set by the National Bank of Ethiopia in 2017 is used for the analysis purpose), c is the number of interest payments (monthly based), t is the storage duration. Therefore, the difference between the final and initial GM would be the OCC for each storage method.

Finally, we derived the net gross margin (NGM) for each storage technique, which was used for MRR analysis.

According to Jones et al. (2014), the percentage returns (%R) to PICS bags storage could be calculated as:

(8) $$\%R=\frac{GM_{t}-\left[1+r_{t}\right]\left(GM_{0}+c\right)}{GM_{0}+c}$$

(9) $$r_{t}=t\text{*r}$$

Moreover, according to Moussa et al. (2014), the additional net cash flow (NCF) (US$ 100 kg^−1^) as a result of using PICS bags per household could be calculated as:

(10) $$NCF=M_{t}-M_{0}-c$$

where %R is percentage returns to PICS bags, $GM_{t}$ is gross margin at the end of storage duration (US$ 100 kg^−1^), $GM_{0}$ is gross margin in the immediate sale after harvest (US$ 100 kg^−1^), c is the material cost of PICS bags (US$), $r_{t}$ is the time adjusted OCC, t is the storage duration (year), r is the annual interest rate (%), NCF is the net cash flow, $M_{t}$ is market price value of the product (US$ 100 kg^−1^) at the end of storage period, $M_{0}$ is market price value of the product (US$ 100 kg^−1^) at the time of harvest.

#### 3.4.3 Econometrics model specification

Binary econometric models (e.g. probit, logit) are usually applied to model household's independent binary decisions, in this case, to use (or not) a single maize storage type. However, in our research location, small-scale farmers use a combination of different storage techniques to reduce storage loss at a given point in time. Likewise, the nature of maize storage techniques is not mutually exclusive, and the adoption process was partial. This means that farmers' decisions to use different maize storage techniques have interrelated characteristics, so univariate modelling was not applicable in this case. Therefore, multivariate model was used to consider for the codependent as well as coinciding aspects of farmers' choices (Greene, 2003). The choice of storage technique was not mutually exclusive that represented by a dummy response (yes/no) function. Moreover, the dummies' responses could be modelled simultaneously by considering the error terms' correlations at the same time (Wooldridge, 2010), and results efficient parameter estimations better than univariate methods (Marra et al., 2017). Therefore, according to Cappellari and Jenkins (2003), the model could be specified in such a way that coincides with multivariate probit (MVP) models for maize storage techniques:

$$y_{ip}^{*}=\beta_{p}^{\prime }x_{ip}+\varepsilon_{ip}\text{,}\;p=1,\;2,\;3$$

$$y_{im}=1\;if\;y_{ip}^{*}>0\;\text{and}\;0\;\mathrm{otherwise}\text{,}$$

where $y_{ip}^{*}$ is farmer's unobserved preferences representing the latent utility of *i* on maize storage technique *p*, $\beta_{p}^{\prime }$ is a vector of unknown coefficients that reflects the effect of changes in the vector of explanatory variables $x_{i}$ on the farmer's preference toward the maize storage technique *p*, $x_{ip}$ is the vector of observed variables that are expected to explain each type of maize storage technique, $\varepsilon_{ip}$ is one of a set of error terms following a multivariate normal distribution, each with a mean of zero and a variance-covariance matrix with values of 1 on the leading diagonal and non-zero correlations on the off-diagonal elements.

##### 3.4.3.1 Dependent variable

The dependent variable is the household decision to use a maize storage technique (Table 1). It is a dummy variable taking the value 1 if the household decides to use a particular maize storage technique and 0 otherwise. In this case, three dependent variables, i.e. household's decision to use NS, TSP, and PICS are modelled jointly by the MVP econometric model. Additionally, for only a robustness check, those three independent variables are modelled independently by a binary logistic regression econometric model, and the results are attached to the annex (Appendix Table 1).

**Table 1:** Descriptions and summary statistics of variables used in the analysis.

Variables	Description of the variables (unit)	Mean	SD
*Dependent*			
**NS**	No storage or sold immediately after harvest (1 = yes, 0 = otherwise)	0.196	0.397
**TSP**	Traditional storage with pesticide (1 = yes, 0 = otherwise)	0.878	0.328
**PICS**	Purdue Improved Crop Storage (1 = yes, 0 = otherwise)	0.667	0.472
*Independent*			
**Age**	Age of the household head (years)	43.90	9.23
**Sex**	Sex of the household head (1 = female, 0 = male)	0.073	0.261
**Labor**	Active labor force (number)	5.65	1.85
**Education**	Educational status of the household head (1 = literate, 0 = otherwise)	0.756	0.430
**Farming experience**	Farming experiences of the household head (years)	24.33	9.87
**Cooperatives**	Cooperatives membership status (years since membership)	14.14	8.97
**Land**	Owner-operated landholding size (ha)	1.76	0.95
**Livestock**	Livestock holding size (TLU)	5.76	2.86
**Crop income**	Annual crop income (US$)	1901.77	1754.23
**Maize production**	Annual volume of maize production (quintal)	45.94	29.10
**Expenditure**	Non-food items expenditure (US$)	1194.47	1038.30
**Input demand**	Farmers reported increased demand for other agricultural inputs (1 = yes, 0 = otherwise)	0.951	0.216
**Extension information**	Access to extension information about improves storage techniques (1 = yes, 0 = otherwise)	0.456	0.499
**Cost of PICS**	Farmers perceived PICS bags needs higher initial cost (1 = yes, 0 = otherwise)	0.76	0.428
**Storage capacity of PICS**	Farmers perceived PICS bags has a higher storage capacity (1 = yes, 0 = otherwise)	0.578	0.495

Where: SD: Standard Deviation; TLU: Tropical Livestock Unit; Quintal: 100 kg.

##### 3.4.3.2 Independent variables

For maize growers to use maize storage techniques as a strategy to reduce storage loss, they need to decide whether the economic benefits exceed the short- and long-term costs. Given this consideration and based on the results of our preliminary survey and review of literature (Adetunji, 2009a; Sekumade and Oluwatayo, 2009; Ibro et al., 2014; Moussa et al., 2014), the decision to use storage techniques was hypothesized to be influenced by a combined effect of various factors including socioeconomic characteristics (e.g., age, sex of the household, active labor force, education, farming experience, cooperative membership experience, resource endowment, expenditure on non-food items, and other factors), institutional and communication conditions (agricultural input demand, access to extension information), and farmers perceived values of the initial price and storage capacity of PICS. For the purpose of this study, explanatory variables influencing the decision to use PICS were given the most attention because our focus was on the economics of the hermetic storage technique.

## 4 Results

### 4.1 Characteristics of the respondents

Descriptions and summary statistics of the variables included in the model are presented in Table 1. TSP and PICS were the most used forms of storage, with 87.8% and 66.7% of respondents, respectively. The mean annual maize production obtained per household was 4.594 t. More than 95% of the respondents used inputs such as fertilizer and improved seed. Only 45.6% of farmers had access and utilized extension information related to PICS. More than 76% of respondents perceived that the price of the PICS bag was high, but 57.8% believed that PICS has the greatest storage capacity as compared to other existing storage techniques.

### 4.2 Farmers’ awareness about maize storage damage and the use of PICS bags

More than 54% of the respondents acknowledge maize damage caused by insect pests (51.3%) and rodents (36%) during storage (Table 2). Information obtained during FGDs showed that the occurrence of pests and rodents has been highly associated with changes in weather conditions such as drought. Farmers reported using a combination of different coping strategies, including insecticide (50.7%), PICS (36.9%), cats (28.9%), rodenticides (23.3%), and selling the crop immediately after harvest (7.6%).

**Table 2:** Occurrence of maize grain storage damage.

Description	Category	Frequency (*N* = 450)	%
**Occurrence of maize grain storage damage**	no damage	203	45.1
	damage	247	54.9
**Reasons for maize grain storage damage**	insect pests	231	51.3
	rodents	162	36
**Coping strategies employed by farmers who encountered maize grain storage damage**^a^	insecticide	228	50.7
	rodenticide	105	23.3
	PICS	166	36.9
	cultural methods (cats)	130	28.9
	selling at harvest	34	7.6

a More than one reason and/or coping strategy can apply.

There was an increase in awareness of PICS over time: 30.2% in 2014, 59.4% in 2015 and 94.2% in 2017 (Table 3). Almost all respondent (99.5%) learned about PICS from extension workers. About two-thirds (66.7%) of the respondents used PICS as a maize storage technique; and of those, 59.3% started in 2015, 33% in 2014, and 7.7% in 2013 (Table 3). The remaining 33.7% did not use PICS. A small proportion of respondents (8.7%) experienced challenges while using PICS, including rodent attacks (6.7%), the need for large storage space (3.7%), and damaged liners (0.7%). Almost all respondents (91.3%) reported no problems with the use of PICS.

**Table 3:** Awareness, utilization and challenges in using PICS.

Description	Category	Frequency	%
**Awareness about PICS (*N = 450*)**			
**Awareness of PICS**	no	26	5.8
	yes	424	94.2
**Initial year of awareness**	2015	252	59.4
	2014	128	30.2
	2013	39	9.2
	2012	5	1.2
**Source of awareness**	MPCSs	2	0.5
	extension workers	422	99.5
**Years of PICS utilization and related problems (*n = 300*)**			
**First year of utilization**	2015	178	59.3
	2014	99	33.0
	2013	23	7.7
**Occurrence of problems with PICS**	no	274	91.3
	yes	26	8.7
**Type of problem**	attacked by rodents	20	6.7
	needs large space	11	3.7
	internal plastic easily damaged	2	0.7

Where: MPCSs: multipurpose cooperative societies.

### 4.3 Economic costs and benefits of maize storage techniques

#### 4.3.1 Gross margin analysis

To determine the profitability of storage, we first assessed the specific variable costs incurred by maize farmers in the study area (Table 4). Purdue Improved Crop Storage had the highest average material cost per unit storage (US$1.73 for a 100 kg capacity bag). On average, a household paid US$3.84 to treat 100 kg of maize with pesticides, which is the cost of about two PICS bags. No storage (selling immediately after harvest) incurred highest transportation cost (US$0.80 100 kg^−1^), followed by TSP (US$0.41 100 kg^−1^) and PICS (US$0.36 100 kg^−1^). The loading/unloading cost varied from US$0.33 100 kg^−1^ to US$0.28 100 kg^−1^ for NS and PICS, respectively. PICS had the highest cost of time wastage at US$0.43 100 kg^−1^, and NS had the lowest (US$0.37 100 kg^−1^). Traditional storage with pesticide incurred the highest cost of dry weight loss (US$1.03 100 kg^−1^) relative to the other methods. Traditional storage with pesticide had the cost of storage pest damage at US$2.27 100 kg^−1^. Overall, TVC was lowest for NS and PICS and much higher for TSP (Table 4). PICS had the highest NGM (US$19.31 100 kg^−1^), followed by NS (US$15.89 100 kg^−1^) and TSP (US$10.83 100 kg^−1^).

**Table 4:** Estimated variable costs for each type of maize storage technique.

Description	Maize storage technique		
	NS (*n* = 88)	TSP (*n* = 395)	PICS (*n* = 300)
**Average duration of storage (months)**	–	5.4	9.6
**Average selling price (US$ 100 kg**^**−1**^**)**	17.81	20.19	24.56
**Average material cost (US$ 100 kg**^**−1**^**)**	0.41	0.41	1.73
**Average chemical cost (US$ 100 kg**^**−1**^**)**	–	3.84	–
**Average transportation cost (US$ 100 kg**^**−1**^**)**	0.80	0.41	0.36
**Average loading and unloading cost (US$ 100 kg**^**−1**^**)**	0.33	0.32	0.28
**Average cost of time wasted during selling (US$ 100 kg**^**−1**^**)**	0.37	0.38	0.43
**Average cost of dry weight loss (US$ 100 kg**^**−1**^**)**	–	1.03	–
**Average cost of storage pest damage (US$ 100 kg**^**−1**^**)**	–	2.27	–
**Total variable cost (US$ 100 kg**^**−1**^**)**	1.91	8.65	2.79
**Gross margin (GM, US$ 100 kg**^**−1**^**)**	15.89	11.54	21.77
**Opportunity cost of capital (US$ 100 kg**^**−1**^**)**	–	0.71	2.46
**Net gross margin (NGM, US$ 100 kg**^**−1**^**)**	15.89	10.83	19.31

Where: NS: no storage or immediate sales; TSP: traditional storage technique with pesticide; PICS: Purdue Improved Crop Storage; -: zero value.

#### 4.3.2 Partial budget analysis

Table 5 provides partial budget results for maize growers. The difference in GM when a household changes from NS to TSP and PICS was US$–4.36 100 kg^−1^ and US$5.87 100 kg^−1^, respectively. The difference for the change from TSP to PICS was US$10.23 100 kg^−1^.

**Table 5:** The estimated partial budget analysis for each storage technique.

Changing storage	Positive effect (US$)			Negative effect (US$)			Total A minus Total B
	AI	DC	Total A	DI	AC	Total B	
**from NS to TSP**	20.19	1.91	22.1	17.81	8.65	26.46	−4.36
**from NS to PICS**	24.56	1.91	26.47	17.81	2.79	20.6	5.87
**from TSP to PICS**	24.56	8.65	33.21	20.19	2.79	22.98	10.23

Where: AI: added income; DC: Decreased cost; DI: Decreased income; AC: Added cost; NS: no storage or immediate sales; TSP: traditional storage technique with pesticide; PICS: Purdue Improved Crop Storage.

In the marginal analysis, only NGM and TVC were used for the estimation of the MRRs. The MRR of shifting from one storage type to the other type is displayed in Table 6. Based on the results, changing from NS to TSP is not recommended because its MRR is not only the lowest, it has a negative value (−0.747). The %R as a result of using PICS bags is estimated 15%, which indicated that the PICS bags return is better than the expected benefits of no storage or selling immediately after harvest. Moreover, the additional net cash flow per PICS bags and at household level is estimated US$5.02 100 kg^−1^ and US$11.58 100 kg^−1^, respectively.

**Table 6:** Estimated marginal rate of return analysis for each type of maize storage technique.

Description	Maize storage technique		
	NS (*n* = 88)	TSP (*n* = 395)	PICS (*n* = 300)
**Average selling price (US$ 100 kg**^**−1**^**)**	17.81	20.19	24.56
**Average total variable cost (TVC, US$ 100 kg**^**−1**^**)**	1.91	8.65	2.79
**Net gross margin (NGM, US$ 100 kg**^**−1**^**)**	15.89	10.83	19.31
**Change in NGM between two consecutive techniques (US$ 100 kg**^**−1**^**)**		−5.06	3.42
**Change in TVC between two consecutive techniques (US$ 100 kg**^**−1**^**)**		6.77	1.07
**Marginal rate of return**		−0.747	3.196

Where: NS: no storage or immediate sales; TSP: traditional storage technique with pesticide; PICS: Purdue Improved Crop Storage.

### 4.4 Econometrics model result

The model results of the determinants of maize growers' decision to use storage techniques are presented on Table 7. The correlations among the error terms (ρ) were statistically significant (prob > chi^2^ < 0.0001), which confirmed our choice to use a MVP model by taking consideration of the interdependent features of farmers’ decision to use multiple maize storage techniques at a time. A binary logistic regression was performed to check the robustness of the results (Appendix Table 1). Most estimated parameters of the MVP and binary logistic regression models have consistent outcomes, evidencing their robustness. However, the result and discussion in the next section are based on the results of the MVP model.

**Table 7:** MVP model results of factors affecting households’ use of maize storage techniques.

Variables	NS	TSP	PICS
**Age**	0.015 (0.014)	0.038* (0.019)	−0.014 (0.014)
**Sex**	−0.277 (0.300)	0.116 (0.348)	0.102 (0.251)
**Labor**	−0.093 (0.061)	0.005 (0.077)	0.086 (0.063)
**Education**	−0.113 (0.169)	−0.042 (0.207)	0.480** (0.152)
**Farming experience**	−0.026* (0.013)	−0.038* (0.017)	0.022 (0.013)
**Cooperatives**	0.030** (0.010)	0.006 (0.013)	0.028* (0.012)
**Land**	0.086 (0.105)	−0.344* (0.160)	−0.513*** (0.114)
**Livestock**	0.002 (0.030)	0.050 (0.042)	0.084** (0.031)
**Crop income**	−0.002 (0.002)	−0.013*** (0.003)	0.016*** (0.005)
**Maize production**	0.001 (0.003)	0.002 (0.005)	−0.011*** (0.003)
**Expenditure**	−0.002 (0.004)	0.031*** (0.010)	−0.011** (0.004)
**Input demand**	−0.358 (0.332)	0.273 (0.434)	−1.316*** (0.389)
**Extension information**	0.091 (0.152)	−0.211 (0.188)	0.536*** (0.153)
**Cost of PICS**	0.353* (0.174)	0.164 (0.193)	−0.427** (0.160)
**Storage capacity of PICS**	−0.362* (0.146)	−0.543** (0.203)	0.530*** (0.143)
**Constant**	−0.777 (0.530)	0.630 (0.712)	1.149* (0.561)

Wald chi^2^ (45) = 203.11; Prob > chi^2^ = 0.0000; *N* = 450; Likelihood ratio test of $\rho_{21}=\rho_{31}=\rho_{32}=0$, chi^2^ (3) = 23.2862, the values in parentheses are robust standard errors of the estimate.

Where: *P < 0.05; **P < 0.01; ***P < 0.001; NS: no storage or immediate sales; TSP: traditional storage technique with pesticide; PICS: Purdue Improved Crop Storage.

Households headed by older farmers were more likely to use TSP (*P* < 0.05). Being-led by literate heads (*P* < 0.01) positively influenced the decision to use PICS, whereas an increase in farming experience had a negative influence on the use of NS and TSP (*P* < 0.05).

Household's years of cooperative membership positively influenced the use of PICS (*P* < 0.05) and NS (*P* < 0.01). Landholding size negatively influenced the decision to use PICS and TSP (*P* < 0.001 and *P* < 0.05, respectively), whereas the livestock holding size positively (*P* < 0.01) contributed to the decision to use PICS. Annual crop income positively influenced the decision to use PICS (*P* < 0.001), in contrast it negatively influenced the decision to use TSP (*P* < 0.001). The probability of using improved agricultural inputs (other than improved maize storage techniques) decreased the likelihood of using PICS (*P* < 0.001). Access to extension information about improved storage techniques positively contributed to the decision to use PICS (*P* < 0.001).

Households who perceived PICS as having a higher initial cost were less likely to use PICS (*P* < 0.01), whereas they were more likely to use NS (*P* < 0.05). Conversely, households who perceived that PICS had a higher storage capacity than the existing storage methods were more likely to use PICS (*P* < 0.001), whereas they were less likely to use TSP and NS (*P* < 0.01 and *P* < 0.05, respectively).

## 5 Discussion

On average, each maize producer in the study used more than one storage technique. The majority of the respondents applied storage chemicals (insecticide and rodenticide) to reduce storage loss. During FGDs and KI interviews, farmers noted that they usually applied pesticides only after grain was heavily infested. Hence, even when chemicals were used, pest damage during storage was likely to be high (Talukder, 2009).

The effectiveness of PICS bags at preventing storage losses has been tested and are now used by farmers in Ethiopia (Kalsa et al., 2019; Alemayehu et al., 2020). In the context of this study, however, the status of using PICS bag was fairly low. On average, only 2.33 PICS bags per household were used; while the maximum individual retention extends to 15 PICS bags per household (Alemu, 2018). Furthermore, participants in the FGDs and KI interviews indicated that this lower use of PICS bags among farmers was related to limited access to finance.

The TSP technique had a long list of variable cost items, with chemical costs accounting for about 44% of the variable costs, an amount that could purchase at least two PICS bags. In Nigeria and Kenya, chemical costs usually account for 12% for one year and about 14% for three years of the total cost of storage (Adetunji, 2009b; Sekumade and Oluwatayo, 2009). The transportation cost dramatically decreases with the increase of storage duration associated with the opportunity costs of transportation service related with time. Because of the timing of the peak production season and selling time of maize, the transportation cost of NS is higher by 95% of TSP and 122% of PICS groups. In the study area, a majority of producers sold their grain during the peak production season to pay their loans for agricultural inputs, thus, the demand for transportation service increases its transportation cost. However, when the storage duration increased, the transportation cost gradually decreased by US$0.05 100 kg^−1^ month^−1^ (Table 5). During the off-production season, and once the farmers’ paid their agricultural inputs loan, no one would be interested in selling their grain, thus, the demand for transportation service decreased. Subsequently, the transportation cost decreased which perhaps shows the time value of storage on decreasing some cost items. The cost of dry weight loss and storage pest damage for TSP was 37% and 81%, respectively, of the TVC of PICS, and no reported costs by the households who stored with PICS bags even though maize was stored for 9.6 months indicating its importance when selecting a maize storage technique. A non-significant dry weight loss by using improved storage method was also recorded in another survey conducted in SSA (Jones et al., 2014), since it has shown to preserve the quality of stored maize grain. In Ethiopia, FAO (2018) estimated the economic loss resulting from pest infestation of stored grain at US$1.6100 kg^−1^.

The selling market price was a major factor driving the differences in GM among storage techniques. The PICS group's selling price after 9.6 months of storage was 38% higher than the NS and 22% higher than the TSP group who stored for 5.4 months. This indicated that the price gain per month of storage is higher for the producers storing with the PICS relative to the other storage methods. Thus, the expected price increment due to longer storage duration would be expected to gain higher GM, besides other factors such as TVC and discount rate. The main reason for the typical price increment was associated with the reduced supply of maize grains to the market during the off-production season. Once the farmers' paid the agricultural inputs loan, they would be less interested to sell their maize grain so that the supply would be decreased. In this case, since maize is a major staple food and consumed by every household regardless of the income level, the market price would be high. Moreover, the higher quality of maize grain stored by PICS was mostly preferred by consumers so that its market price would be high relative to others.

The high TVC ratios of TSP to NS (4.5:1) and PICS (3.1:1) indicate that TSP is not economically viable. The best GM was derived from PICS, which was considered the most profitable choice in this analysis. This result is corroborated by the qualitative and quantitative insights of studies that show the profitability of PICS bags in SSA (Baributsa et al., 2014; Ndegwa et al., 2016; FAO, 2018).

The partial budget analysis results showed that maize growers can change to any storage techniques except TSP, because it had a negative GM in all scenarios. A change to PICS had the greatest (positive) difference. Moreover, the MRR ratio was 3.196 when the storage technique shifted from NS to PICS, which is consistent with the partial budget results. The partial budget and MRR results are consistent with most research results conducted in other areas in SSA, with most showing that hermetic bags have the highest GM and MRR ratios (Adetunji, 2009b; Sekumade and Oluwatayo, 2009; Jones et al., 2011). The net cash flow result told us a household could gain an additional income of US$5.02 100 kg^−1^ as a result of using PICS bags. Overall, the advantage of the PICS storage is driven by the assumption that price increases with storage, and increases more rapidly as the storage period increases. The values of US$6.75 100 kg^−1^ and US$4.37 100 kg^−1^ have been obtained on PICS bags relative to the price of NS and TSP, respectively. Moreover, the advantages of having fewer variable costs except for the initial material cost also contributed to the highest net revenue.

The econometric model results implied that farmers’ decisions to use different maize storage techniques were significantly influenced by various socioeconomic factors, extension and communication approaches, and their perceptions about the PICS technology. Elderly households were more likely to use TSP maize storage method; most likely because they are reluctant to use new and modern storage techniques. Similarly, literate households were more likely to use PICS. This result is plausible possibly because they might have had access of information which led to a better understanding of the benefits it offered and the opportunity costs of storage loss. It is generally believed that being literate will help farmers in comprehending the dynamics and benefits of improved postharvest management, further enhancing their ability to efficiently manage the adopted storage technology (PICS). A study conducted in 10 countries in West and Central Africa indicated that having a formal education did not have an impact on adoption of PICS bags, except in Burkina Faso (Moussa et al., 2014). The latter contrasting finding of Moussa et al. (2014) in Burkina Faso (negative effect) might be explained by other factors (e.g., personal behavior, culture, gender roles, ethnic group, etc.) (Mercer, 2004), which are beyond the scope of this study. More experienced farmers were less likely to use NS and TSP than those with less experience. This was likely due to their farming experience which helped them to understand the change in market prices immediately after selling harvest with NS, and the degree and extent of storage losses and the costs incurred with TSP.

Cooperative members were more likely to use PICS technology. This presumably highlights the importance of agricultural cooperatives in promoting the adoption of improved postharvest storage technologies. Currently, agricultural inputs, including storage techniques, are being disseminated to farmers through rural cooperatives. Membership in these organizations helps increase farmers’ awareness about storage loss and benefits of storage through training and demonstrations (FAO, 2018). Likewise, awareness about improved storage techniques through extension information increased the likelihood of using PICS bags. This result was in agreement with the finding of Omotilewa et al. (2019), who asserted that extension information provided to farmers should include benefits of hermetic bags such as eliminating the use of chemicals and problem of development of resistance to pesticides, the simplicity of use, and quality of the stored grain. Ultimately, these benefits may encourage farmers to choose PICS bags from other grain storage technologies. However, limited availability of PICS bags and timely information on the technology are some of the challenges for adoption (Moussa et al., 2014; Nouhoheflin et al., 2017).

Contrary to our expectation, households with a larger tract of land were less likely to use TSP and PICS. Farmers with larger land size are expected to have a high volume of production of different crop types. In this case, farmers tend to use two options. First, they may use other storage options with larger storage capacity, other than PICS. Second, they may sell at or soon after harvest. This result suggests that there is a need to introduce postharvest solutions for larger scale producers (e.g. larger silos) which was also noted by FGDs and KI participants. On the other hand, households with more livestock were more likely to use PICS than other storage techniques. This result holds true because farmers with more livestock have greater financial capacity from selling livestock or their byproducts (e.g. milk), which may enable them to absorb costs associated with adopting new technologies (Alemu, 2014). Moreover, owning more livestock would allow farmers to partly substitute their chemical fertilizer application by organic manure (Nigussie et al., 2017). As a result, reduced demand for chemical fertilizer would in turn enhance their savings. Logically, increased savings would encourage farmers’ investment on PICS bags, allowing them to store higher quality maize to sell later at higher prices. The inverse relationship between annual volume of maize produced and the use of hermetic technology could be due to the amount of money required to purchase the number of PICS bags to store large quantity of grain. In addition, labor and time required for bagging has also negative influence on farmers' tendency to use the PICS bags when large quantities are involved. Thus, for such large volume of production, they may need larger sized containers instead.

Surprisingly, an increased access to and utilization of agricultural inputs other than improved maize storage reduced the tendency of farmers to use PICS bags, most likely by reducing the amount of income available for other expenditures. More specifically, these farmers were more likely to pay for improved agricultural inputs that would increase their production before deciding to buy PICS bags. In addition, these farmers were more likely to sell a significant portion of their production immediately after harvest and prioritize paying off agricultural input loans rather than investing in purchasing PICS bags. Furthermore, the model results implied that farmers’ perception of the high price of PICS bags negatively affected their decision to use the technology. Nouhoheflin et al. (2017) also noted that the initial investment was the challenge in the adoption of the PICS bags. Likewise, farmers cited cost (price too high or expensive) as a key constraint of using PICS bags in West and Central Africa (Moussa et al., 2014), Niger, Burkina Faso and Nigeria (Ibro et al., 2014), and Kenya (Baributsa and Njoroge, 2020).

## 6 Conclusions

This study is aimed to estimate the costs and economic benefits of alternative maize storage techniques and analyze the factors influencing maize growing farmers' decisions to use them in northwestern Ethiopia. The results showed that some farmers sell their grain immediately after harvest because they need cash to repay agricultural input loans; and others sell due to lack of good storage methods. For small-scale farmers who store, they lose substantial quantities of maize because of damage caused by insects and other pests. Traditional storage with pesticide has been the most predominant maize storage technique used by small-scale farmers in Ethiopia. However, unsafe use of chemicals pose major health hazards in Ethiopia (Negatu et al., 2016).

Hermetic bags, being promoted in Ethiopia, are viable alternatives to traditional methods and insecticides that are commonly used by farmers. Small-scale farmers are more likely to invest in PICS bags when they understand the benefits of the technology. There is need to continue with creating awareness and demonstrations to drive the demand for improved grain storage technologies. More specifically, technology promotional activities should target farmers with larger livestock, cooperative members, literate, and experienced ones. Moreover, improving the availability of PICS bags and the efficiency of the supply chain could help increase its access and adoption. Introducing hermetic storage solutions with bigger capacity would help reduce storage losses among farmers producing large volume of maize. In addition, there is a need for more research on the health and environmental benefits of using hermetic bags.

## CRediT authorship contribution statement

**Gashaw Tenna Alemu:** Conceptualization, Methodology, Formal analysis, Investigation, Data curation, Writing - original draft. **Zerihun Nigussie:** Methodology, Validation, Formal analysis, Resources, Writing - review & editing. **Nigussie Haregeweyn:** Methodology, Validation, Resources, Writing - review & editing. **Zewdu Berhanie:** Methodology, Validation, Resources, Writing - review & editing. **Beneberu Assefa Wondimagegnehu:** Methodology, Validation, Resources, Writing - review & editing. **Zemen Ayalew:** Methodology, Validation, Resources, Writing - review & editing. **Dessalegn Molla:** Methodology, Validation, Resources, Writing - review & editing. **Eric Ndemo Okoyo:** Methodology, Validation, Resources, Writing - review & editing. **Dieudonne Baributsa:** Methodology, Validation, Resources, Writing - review & editing.

## Declaration of competing interest

The authors declare the following financial interests/personal relationships which may be considered as potential competing interests: Dieudonne Baributsa is a co-founder of PICS Global Inc., a social enterprise that commercializes post-harvest technologies (including PICS bags) to smallholder farmers across the world and hence declares a conflict of interest. The other authors declare no conflict of interest. The funder (BMGF) had no role in the design of the study; in the collection, analyses, or interpretation of data; in the writing of the manuscript, or in the decision to publish the results.

## Figures and Tables

**Appendix Table 1:** Binary logistic regression analysis of factors affecting household's use of each maize storage technique.

Variables	NS	TSP	PICS
**Age**	0.026 (0.024)	0.079* (0.039)	−0.018 (0.024)
**Sex**	−0.496 (0.559)	0.267 (0.746)	0.059 (0.444)
**Labor**	−0.163 (0.106)	0.030 (0.157)	0.127 (0.116)
**Education**	−0.200 (0.298)	−0.019 (0.396)	0.872** (0.283)
**Farming experience**	−0.042 (0.022)	−0.070 (0.037)	0.034 (0.021)
**Cooperatives**	0.050** (0.018)	0.013 (0.027)	0.053** (0.020)
**Land**	0.139 (0.188)	−0.753* (0.303)	−0.980*** (0.201)
**Livestock**	0.006 (0.053)	0.068 (0.080)	0.139* (0.057)
**Crop income**	−0.004 (0.005)	−0.015** (0.006)	0.040*** (0.010)
**Maize production**	0.001 (0.006)	0.001 (0.008)	−0.018** (0.006)
**Expenditure**	−0.005 (0.008)	0.063** (0.021)	−0.0229** (0.008)
**Input demand**	−0.562 (0.590)	0.218 (0.832)	−2.329** (0.750)
**Extension information**	0.161 (0.272)	−0.524 (0.362)	0.842*** (0.262)
**Cost of PICS**	0.632 (0.327)	0.311 (0.427)	−0.697* (0.288)
**Storage capacity of PICS**	−0.621* (0.262)	−1.066* (0.427)	0.950*** (0.250)
**Constant**	−1.339 (0.916)	0.908 (1.350)	1.728 (1.075)
**N**	450	450	450
**Wald chi2 (15)**	33.56	52.57	80.96
**Prob > chi**^**2**^	0.0039	0.0000	0.0000
**Pseudo R**^**2**^	0.0653	0.2232	0.3301

The values in parentheses are robust standard errors of the estimate. Where: *P < 0.05; **P < 0.01; ***P < 0.001; NS: no storage or immediate sales; TSP: traditional storage technique with pesticide; PICS: Purdue Improved Crop Storage.

## Footnotes

1 An ordinary polypropylene bag which is available in the local market.

2 A local storage bag which is made from cattle hide and skin.

3 An ordinary storage bag which is made from fibre.
